# Tissue Regeneration of Radiation-Induced Skin Damages Using Protein/Polysaccharide-Based Bioengineered Scaffolds and Adipose-Derived Stem Cells: A Review

**DOI:** 10.3390/ijms26136469

**Published:** 2025-07-04

**Authors:** Stefana Avadanei-Luca, Isabella Nacu, Andrei Nicolae Avadanei, Mihaela Pertea, Bogdan Tamba, Liliana Verestiuc, Viorel Scripcariu

**Affiliations:** 1 Faculty of General Medicine, “Grigore T. Popa” University of Medicine and Pharmacy, 700115 Iasi, Romania; stefana.luca@d.umfiasi.ro (S.A.-L.); andrei.avadanei@umfiasi.ro (A.N.A.); mihaela.pertea@umfiasi.ro (M.P.); bogdan.tamba@umfiasi.ro (B.T.); viorel.scripcariu@umfiasi.ro (V.S.); 2 Department of Plastic Surgery and Reconstructive Microsurgery, “St. Spiridon” Emergency County Hospital, 700111 Iasi, Romania; 3 Faculty of Medical Bioengineering, “Grigore T. Popa” University of Medicine and Pharmacy, 700115 Iasi, Romania; nacu.isabella@icmpp.ro; 4 Department of Vascular Surgery, “St. Spiridon” Emergency County Hospital, 700111 Iasi, Romania; 5 Center for Advanced Research and Development in Experimental Medicine (CEMEX), “Grigore T. Popa” University of Medicine and Pharmacy, 700115 Iasi, Romania; 6 Department of Surgery, Regional Institute of Oncology, 700483 Iasi, Romania

**Keywords:** protein/polysaccharide scaffolds, adipose-derived stem cells, skin regeneration, radiotherapy, side effects

## Abstract

Radiation therapy, a highly effective cancer treatment that targets cancer cells, may produce challenging side effects, including radiation-induced skin tissue injuries. The wound healing process involves complex cellular responses, with key phases including hemostasis, inflammation, proliferation, and remodeling. However, radiation-induced injuries disrupt this process, resulting in delayed healing, excessive scarring, and compromised tissue integrity. This review explores innovative approaches related to wound healing in post-radiotherapy defects, focusing on the integration of adipose-derived stem cells (ADSCs) in protein/polysaccharide bioengineered scaffolds. Such scaffolds, like hydrogels, sponges, or 3D-printed/bioprinted materials, provide a biocompatible and biomimetic environment that supports cell-to-cell and cell-to-matrix interactions. Various proteins and polysaccharides are discussed for beneficial properties and limitations, and their compatibility with ADSCs in wound healing applications. The potential of ADSCs-polymeric scaffold combinations in radiation-induced wound healing is investigated, alongside the mechanisms of cell proliferation, inflammation reduction, angiogenesis promotion, collagen formation, integrin binding, growth factor signaling, and activation of signaling pathways. New strategies to improve the therapeutic efficacy of ADSCs by integration in adaptive polymeric materials and designed scaffolds are highlighted, providing solutions for radiation-induced wounded skin, personalized care, faster tissue regeneration, and, ultimately, enhanced quality of the patients’ lives.

## 1. Introduction

Radiotherapy is a highly effective cancer treatment that targets cancer cells by depositing high physical energy of the radiation into malignant tumors. More than 50% of patients are treated using radiotherapy, either as a singular therapy or in combination with chemotherapy or surgery [1]. This medical procedure, known for its curative potential and palliative relief, operates by damaging cancer cell DNA [2], preserving healthy tissue. Various radiotherapy intensities and techniques respond to the patient’s needs and deliver the radiation to tumor lesions with great precision [3].

Despite its therapeutic promise, radiotherapy can trigger complex tissue responses and side effects, from acute skin reactions to long-term complications, such as radiation-induced neoplasms and fibrosis. These effects are closely related to radiation dose, tissue irradiation, and the time interval between treatments [4].

Significant advancements have been registered in the standardization and precision of radiation therapy, and, with all that, the incidence of radiation-induced skin injury is gradually increasing. More than 85% of tumor patients have developed various skin damages due to radiotherapy, from erythema and peeling to ulcers or even necrosis of skin tissue [5]. Radiation-induced skin damage is characterized by disruptions in wound healing processes, microvascular damage, and alterations in cell communication. The complex phases of skin wound healing involve hemostasis, inflammation, proliferation, and remodeling, all tightly regulated by cellular signaling molecules and specific growth factors [6,7].

In response to these challenges, regenerative medicine and tissue engineering have emerged as promising treatments in radiation-induced skin injuries. The tissue engineering field, included for the first time 25 years ago by Langer and Vacanti, combines engineering design with biological mechanisms to regenerate or replace the wounded tissue through a synergistic effect of the biomaterials (as scaffolds with controlled morphology and biodegradability), biological molecules, and many cell types, including stem cells, based on their ability to differentiated into any cell of an organism and self-renewal capacity [8]. Adipose-derived stem cells (ADSCs), known for their pluripotency and proliferative capacity, paracrine effects, and low immunogenicity, are under the spotlight. Moreover, adipose tissue is at the top of the list of stem cell sources, due to its abundance, its accessibility, and its having a less painful procedure for cell collection when compared to other tissues [9]. Combined with polymeric bioengineered scaffolds, ADSC-based therapies have shown enhanced wound healing potential, promoting tissue regeneration and closure [10].

Bioengineered scaffolds, such as hydrogels and nanofibers, micro- and nanoparticles, have demonstrated good results in managing radiation-induced injuries, owing to their mechanical and biochemical properties [11,12]. The synergy between ADSCs and these scaffolds promotes a stimulating microenvironment for tissue repair, improving cell-to-cell and cell-to-matrix interactions [13,14].

In order to obtain more effective wound healing, the treatments are continuously refined, from modern dressings to stem cell therapies and tissue-engineered skin substitutes [15,16]. Moreover, an emergent technology, as 3D bioprinting, that integrates additive manufacturing with biological sciences, permits the mimicking of the morphology and functionality of native biological tissues. Bioprinting has the capacity to print living cells and biomaterials and offers new capabilities for regenerative medicine [17]. As research in this domain progresses, it could transform the management and healing of once-daunting wounds, outlining a more efficient tissue recovery.

This review aims to critically examine the current state of ADSCs-based therapies combined with protein/polysaccharide scaffolds for the treatment of radiation-induced skin damage. ADSCs’ advantages, types of proteins/polysaccharides, and their properties compared to synthetic polymers are discussed, as well as their composition and architecture, preparation methods, biological mechanisms, and clinical implications.

## 2. Radiotherapy: Between Treatment and Its Side Effects

### 2.1. Radiotherapy in Cancer Treatment

Radiotherapy (RTX), also known as radiation therapy, is one of the major therapies for cancer treatment. This medical technique uses the power of high-energy radiation to selectively target and obliterate cancer cells or reduce the size of tumors [18]. Radiotherapy is recognized for its efficacy, offering a curative approach in many instances, as well as serving a valuable role in conjunction with other treatments like surgery, chemotherapy, and immunotherapy. Furthermore, it often serves as a palliative solution, providing relief from distressing symptoms for patients with advanced-stage cancers. Radiotherapy functions by damaging the DNA within cancer cells, thereby preventing their uncontrolled growth and division [19]. The process involves the precise delivery of ionizing radiation to the tumor site while minimizing exposure to surrounding healthy tissue. Ionizing radiation can be electromagnetic or particulate. Electromagnetic radiation involves high-energy photons in the form of X-rays and gamma rays. X-rays are produced externally by electrical machines, while gamma rays are produced by the decay of radioactive isotopes, such as cobalt 60 [20]. Radiotherapy intensities refer to the levels of radiation dose and energy used in radiation therapy treatments and include Conventional Radiotherapy (CRT) (low- to medium-energy X-rays, offering fixed radiation dose over several weeks) [21], Intensity-Modulated Radiation Therapy (IMRT) (customized X-ray beam intensities for precise tumor targeting, minimizing exposure to surrounding healthy tissue) [22], and Image-Guided Radiation Therapy (IGRT) (combining X-ray imaging with treatment to visualize tumors in real-time, allowing precise adjustments) [23].

Several techniques and modalities are employed in radiotherapy, such as external beam radiation, brachytherapy (internal radiation), and stereotactic radiosurgery, each tailored to the unique characteristics of the cancer and the patient’s individual needs. However, alongside its remarkable therapeutic potential, radiotherapy presents challenges and side effects [24]. The side effects can vary in type, severity, and duration, depending on factors such as the location of the tumor, the dose of radiation, and the individual patient’s sensitivity. Common acute side effects include skin reactions such as radiation dermatitis (erythema, dryness, irritation, blistering, or peeling) [25], fatigue [26], gastrointestinal disturbances (nausea and vomiting, difficulty swallowing or breathing) [27], bowel or bladder changes, and hair loss. Long-term side effects may become evident months or even years after radiotherapy, including radiation fibrosis (stiffness, pain, or reduced organ function in the treated area) [28], calcification in soft tissues [29], secondary cancers (radiation-induced neoplasms) [30], cognitive impairment (especially in pediatric patients), cardiovascular complications, and hormonal changes [31].

The severity of the side effects is directly proportional to the absorbed doses. Other considerations include the area of the irradiated tissue, the radiation type, protraction, and the time gap between radiation and the reported clinical consequences [32].

While the initial side effects of radiation therapy heal naturally, the long-term side effects are unpredictable and frequently distressing. In addition to causing subsequent pain, contractures, and functional impairment, the resulting injury can have severe effects on tissues by causing chronic tissue fibrosis and destruction [33]. Despite significant advancements in radiation therapy technology, non-specific radiation damage to non-cancerous tissues still affects people to various degrees [34]. The most significant challenges in treating radiation-induced damage include the unbalanced inflammatory response, oxidative stress response, lack of angiogenesis, and a high risk of bacterial infection [35].

### 2.2. Radiation-Induced Damages in Skin and Wound Healing

At various points, radiation interferes with the natural process of wound healing [36,37]. Microvascular damages, extracellular matrix alterations, and cellular depletion are pathologic processes that induce local tissue hypoxia [38]. Considering the changes in cellular signaling between dermal fibroblasts, keratinocytes, and immunocompetent cells, exposure to radiation severely damages the skin barrier function, regeneration potential, and capillary integrity [39]. Radiation-induced skin wounds heal more slowly than normal due to an imbalance in the inflammatory response [40]. Free radicals and pro-inflammatory mediators, including interleukins (IL)-1 and IL-6, transforming growth factor (TGF)-1, and connective tissue growth factor (CTGF), are linked to the observed tissue harm; the skin becomes fibrotic, hypoxic, and hypovascular [41].

**Figure 1:**
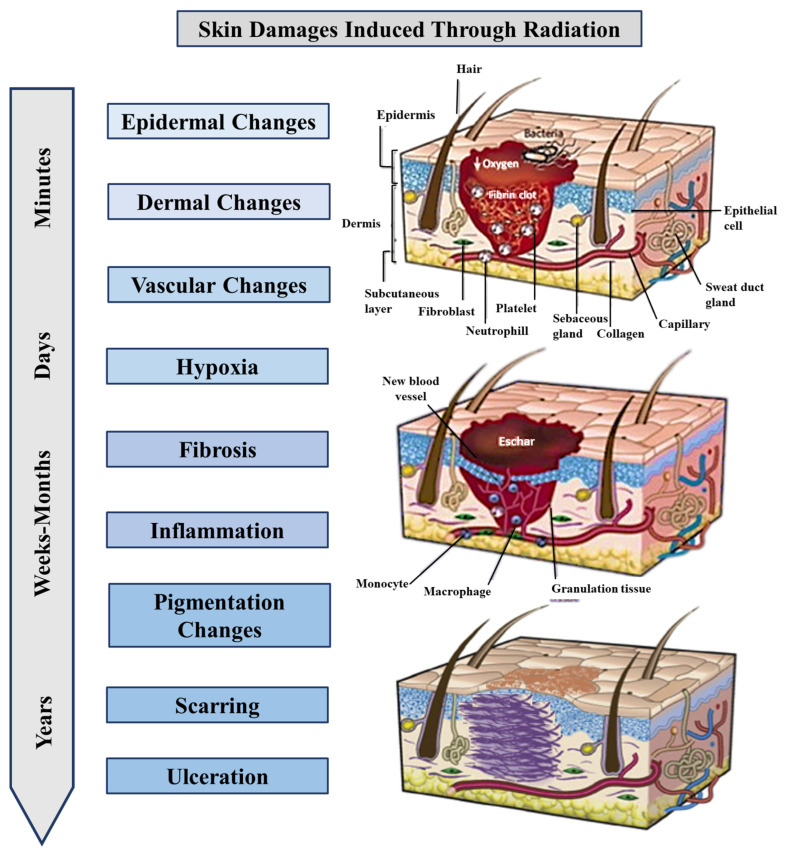
Time-dependent stages in radiation-induced wounds: from epidermal changes to large changes in dermal tissue.

The early inflammatory response in radiation-induced skin damage is primarily controlled and progressively amplified by a number of proinflammatory cytokines (tumor necrosis factor [TNF]-a, interleukins [IL-1, IL-3, IL-5, IL-6), chemokines (IL-8, CC receptor, eotaxin), receptor tyrosine kinase, and adhesion molecules (vascular cell adhesion molecule [VCAM], intercellular adhesion molecule [ICAM]-1, E-selectin) [42]. Radiotherapy will impair wound healing by reducing cell proliferation, damaging blood vessels, and leading to excessive scarring (fibrosis). Angiogenesis, the formation of new blood vessels essential for oxygen and nutrient supply, can be limited due to vascular changes (Figure 1) [36].

Key healing cells like fibroblasts and keratinocytes may experience reduced growth, affecting tissue regeneration and wound closure [36]. Moreover, radiation can trigger excessive collagen production, resulting in scarring or fibrosis that limits tissue flexibility, while the weakened immune responses in irradiated areas raise the risk of infection, further preventing wound healing. Before or after surgery, radiation may delay wound healing, increase the risk of infection, and compromise tissue integrity [43].

Rebuilding skin functions after radiation-induced injuries requires complete skin wound healing that returns full-thickness skin with its appendages, and involves all phases of the skin wound healing process, including hemostasis, inflammation, proliferation, and remodeling [44]. Chemotaxis, phagocytosis, neocollagenesis, remodeling of collagen (Col), and other biological processes involved in these stages are all strictly controlled [45,46,47]. The production of growth factors and cytokines that enhance wound signals and assist the reparative process is important; macrophage accumulation later plays a significant role [48,49,50]. During tissue repair, the temporary fibrin scaffold is replaced by newly deposited extracellular matrix (ECM) [51]. In the proliferative phase, keratinocytes proliferate to seal the wound while myofibroblasts contract to reduce the size of the wound. Endothelial cells contribute to the formation of new blood vessels through angiogenesis, supplying oxygen and nutrients [52].

**Figure 2:**
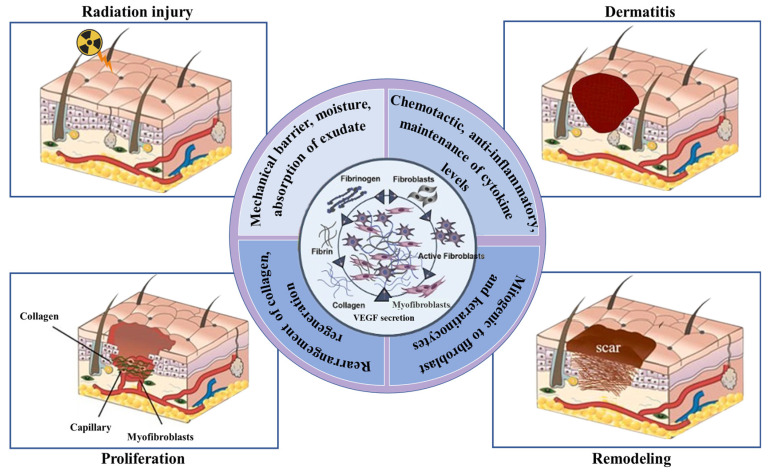
Repair of the radiation-induced wounds and involved biomolecules and processes.

All stages of wound healing (Figure 2) involve the simultaneous proliferation of endothelial cells, which revascularize the injured tissue. Finally, the extracellular matrix (ECM) remodels and produces a scar over weeks to years. The tensile strength of the wound gradually approaches that of uninjured tissue as collagen bundles enlarge. The organization of collagen fibers improves, resulting in a greater contraction. A completely developed scar is characterized by a low number of cells, reduced vascularization, and increased tensile strength due to changes in the density of fibroblasts and macrophages, blood flow, and metabolic activity [53,54,55].

### 2.3. Cells and Skin Regeneration

Regeneration of the skin is a dynamic process, and different cell types contribute to skin repair, including keratinocytes, fibroblasts, and endothelial cells, but also some of the less-studied cell lineages, such as stem cells, adipocytes, melanocytes, and cutaneous nerves [56]. Cells interact through various signaling molecules and growth factors, ensuring a coordinated response and repair of the wounded skin. The cell’s function is determined by the molecular structure and physical characteristics of ECMs, which also act as an instrument for intercellular communication by transmitting mechanical signals [57].

Fibroblasts are large, flat, and elongated (with spindle shapes) cells, generally derived from primary mesenchymal cells, widely distributed in different tissues, and especially in the connective tissue [58]. These cells are metabolically active and are able to continuously synthesize, secrete, and degrade ECM components. Fibroblasts control the tissue architecture and turnover by delivering matrix metalloproteinases (MMPs) and their inhibitors (TIMPs) [59].

In injured skin, fibroblasts became extremely active and differentiated into myofibroblasts, participating in the secretion of ECM components, from collagen and elastin, to glycoproteins, such as fibronectin and laminin, and other molecules involved in the healing process. Both fibroblasts and myofibroblasts support wound healing, the formation of new ECM and collagen structures, and the fibrillar collagen forms releasing in the interstitial matrix of the dermis. Moreover, the cells facilitate the fibrin clot disintegration [60]. The hyperactive myofibroblasts are often associated with excessive synthesis of the matrix proteins and hypertrophic scars formation or scleroderma; however, in severely damaged tissue, the hyperproduction of matrix proteins contributes to the remodeling process [61].

Keratinocytes are the original cells in the epidermis and are major cellular components of this part of the skin. They are involved in recruiting, activating, and stimulating the cells associated with wound healing, with a large contribution to rebuilding the epidermal layer of the skin [58]. Keratinocytes react to the epidermal barrier disordering and release cytokines, signaling molecules for cell activation, migration, and proliferation [62,63]. Moreover, keratinocytes interact with dermal fibroblasts through paracrine signaling, stimulating the differentiation into myofibroblasts and enhancing the capability to produce and deposit ECM components. After full re-epithelialized of the wound, mature keratinocytes release signaling molecules that reduce ECM synthesis and increase its degradation [64].

**Figure 3:**
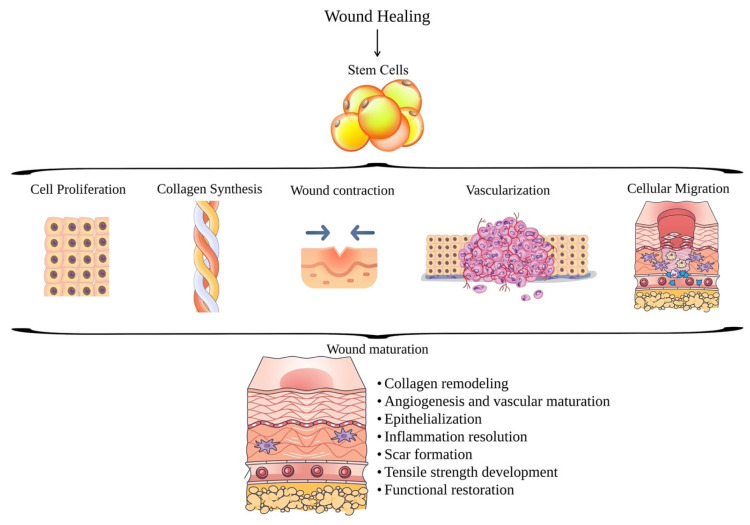
Stem cells in wound healing mechanism, including cell proliferation, collagen synthesis, cell migration, vascularization, and wound maturation.

Various stem cells (SCs) can be involved in repairing the injured tissue (Figure 3) due to their renewal capacity. Adult body tissues and embryos include unspecialized stem cells that may differentiate into various types of cells in the organism and are capable of self-regeneration [65].

The umbilical cord, bone marrow, brain, skeletal muscles, blood, skin, and liver are organ sources of autologous stem cells [66,67]. Adult stem cells are involved in all stages of cutaneous wound healing. Bone marrow-derived stem cells (BMSCs) play an important role in the inflammatory stage, homing to injured tissues before proliferating and differentiating into required lineages. Furthermore, stem cells present in the skin are precursors for mast cells, important directors of the inflammatory phase. However, the division and differentiation of tissue-specific adipocyte stem cells (ADSCs) regenerate damaged or lost tissue in the proliferative phase. Meanwhile, epithelial stem cells from inter-follicular and hair follicle bulge proliferate and differentiate into cell lineages of keratinocytes for re-epithelialization [68]. Likewise, migratory BMSCs may also contribute to fibroblast populations present in wounds [69].

Hematopoietic stem cells (HSCs), derived from bone marrow, play a role in the production of new endothelial cells involved in revascularization. Endothelial progenitor cells (EPCs) are also critical in wound healing due to their effect on the secretion of growth factors [70]. Growth factor signaling is responsible for stem cell response in the wound bed, and a number of different growth factors play important roles in skin wound healing. The expression of HIF-1α is induced by hypoxic wound environments, which upregulates FGF, while HIF-2α upregulates VEGF, which is involved in revascularization. In epithelial wounds, growth factors control keratinocyte migration and ECM remodeling, and wound area filling. Decreased stem cell function was associated with a lack of growth factors in chronic wounds [69,71].

### 2.4. Wound Healing Therapies

Wound healing is a dynamic and complex process that is naturally initiated in the human body after external injuries. However, the chronic or complicated wounds may require specialized interventions to promote efficient tissue regeneration and closure [72]. Advanced dressings, like hydrogels and foams, along with topical agents, such as growth factors, enhance the wound healing process [73]. Bioactive dressings, oxygenation treatment [74], the use of angiogenic growth factors [75], and stem cells are therapeutic approaches for treating refractory wounds. To create this supportive environment for healing, gelling fibers, alginates, and hydrogels with new design and predictable mechanism of action, membranes with silver coatings, and sheets of allogenic skin are now emplored [76], as well as products infused with antibacterial substances, such as silver, polyhexamethyline biguanide, honey, and others [77]. Clinicians have access to a wide range of increasingly sophisticated treatments, including platelet-rich plasma, collagen-derived products, negative pressure wound therapy, skin autograft, matrix metalloproteinases (MMP) inhibitors, artificial cellular and acellular matrices, and collagen-derived products [78,79,80,81]. Over time, skin substitutes, playing the role of tissue-engineered artificial skin equivalents, have been developed as alternatives [82]. Three-dimensional bioprinting and advanced biomaterials create functional skin equivalents to enhance tissue regeneration, and could outperform the skin grafts and become promising therapeutics for cutaneous wounds [83,84,85].

## 3. Bioengineered Scaffolds

**Figure 4:**
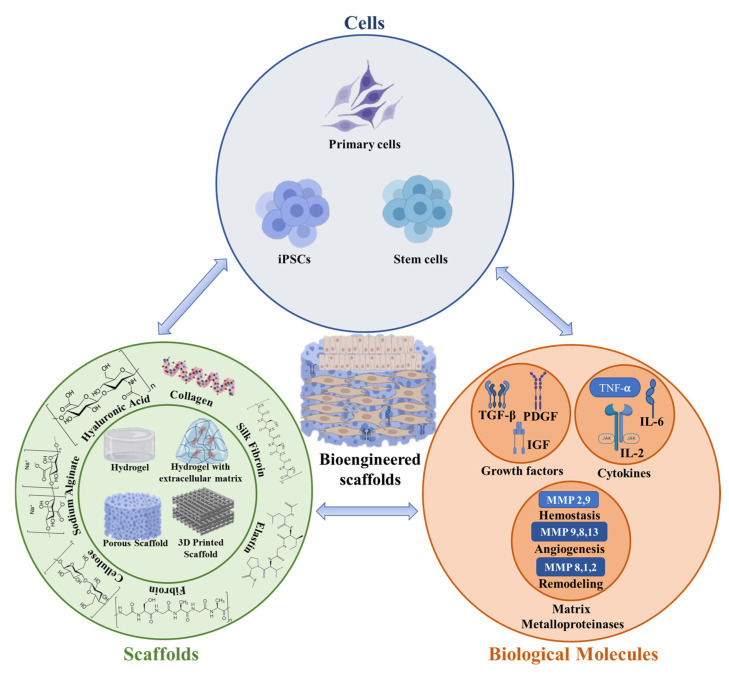
The tissue-engineering triad: protein/polysaccharide-based scaffolds, cells, and biological molecules are used to synergistically repair/regenerate natural tissue.

To enhance any tissue engineering approach, it is essential to choose a suitable cell type and substrate material, and stem cells and polymeric materials are selected because of their distinct features (Figure 4).

Stem cells have the capacity for self-renewal and differentiation into particular cell types in response to suitable signals [86]. Polymeric materials provide suitable physical-chemical and biological properties, are biocompatible, degradable, and amenable to variable processing and property design. Tissue engineering often uses polymers able to stimulate stem cell activity by transmitting physical, chemical, mechanical, and/or biological signals [87]. As an example, fibronectin, a natural polymer, is recognized for its capacity to enhance cell attachment [88]. Some combined polymers or composite scaffolds possess biological, chemical, and mechanical benefits [89,90].

### 3.1. Advanced Polymeric Biomaterials Versus Traditional Technologies

Traditional methods for the clinical treatment of radiation-induced skin damage use the necrotizing skin debridement, topical corticosteroids and antibiotics, or wound dressings [91,92]. Novel strategies have been explored, including hyperbaric oxygen therapy, cytokine therapy, and more advanced techniques, such as stem cell therapy and scaffolds, which mimic the natural extracellular matrix (ECM) and stimulate the cells growth by creating a friendly biological environment [93,94]; such scaffolds/bioscaffolds stimulate the fibroblasts and epithelial cells involved in tissue repair to migrate and proliferate rapidly, and control tissue repair and regeneration [95].

Biomaterials, natural origins, synthetic or acellular tissue matrices, have to meet some requirements in order to substitute the native tissue with a smooth biointegration: to be biocompatible, biodegradable, and bioresorbable, and to have mechanical resistance [96,97]. Woven dressings, porous scaffolds, 3D printing materials, nanofibers, hydrogels, and other types of dressings are among those available at this point to aid wounds heal [98,99]. Natural polymers (e.g., polysaccharides, proteins) are, predictably, the most studied materials in bioengineering scaffolds [100]. Because of their unique features (e.g., antibacterial activity), polysaccharides such as dextran [101], chitosan [102], and hyaluronic acid [103] are widely investigated for cutaneous wound healing.

Compared to natural biomaterials, artificial and synthetic ones (polymer-based or ceramic elements) with favorable mechanical features were explored in wound healing protection [104,105]. Natural and artificial materials each have their own variety of strengths and downsides, and, to take advantage of both of their features, hybrid materials are regularly manufactured, leading to widespread use in the field of cutaneous wound healing [106]. Electrospinning technology, 3D bioprinting technology, microfluidic technology, and biomaterial/stem cell technology are the highlighted technologies in the production of bioengineered scaffolds [55]. Hence, bioactive materials are a promising therapeutic tool that improves upon the drawbacks of current methods while changing the wound microenvironments and triggering certain behaviors in vital cells, thereby shifting the healing process in the desired direction [107].

The field of scaffold-based tissue engineering [108] emerged as a solution to address the drawbacks associated with direct cell suspensions. Its primary objective is to create effective methods for delivering cells and to fabricate advanced three-dimensional (3D) tissue models, biocompatible, biodegradable, and mechanically robust scaffolds [109,110] with a well-connected porosity structure, facilitating effective transportation and exchange of gases and specialized biomolecules [111]. Once integrated into the body, the scaffold have to meet the following applicative needs: (i) serve as a reliable structure for cell adhesion, proliferation, and differentiation as a substrate, (ii) establish the necessary biomechanical conditions for coordinated tissue regeneration, (iii) facilitate the distribution of nutrients and oxygen, and (iv) enable the encapsulation and release of cells along with growth factors [112].

### 3.2. Polymeric Biomaterials for Scaffolds

Polymers are the backbone of the scaffolds, performing an important function in facilitating their functionality. Depending on their nature, they may be natural or synthetic and must have properties such as biocompatibility and biodegradability in order to be processed as scaffolds for substitution of the native tissue without being rejected [93].

#### 3.2.1. Natural Polymers—Proteins and Polysaccharides

Natural polymeric materials possess superior biocompatibility, degradability, and cell recognition characteristics, frequently exploited in the wound healing processes, replacing native ECM structural components and skin cellular background [113]. In addition, naturally derived polymers are abundant in functional groups, easily modified and crosslinked, and are involved in physical and chemical/biochemical stimuli-responsive reactions [114,115,116]. Natural polymers are lengthy chains comprised of repeated covalently bound compounds, such as nucleotides, amino acids, or monosaccharides, and often contain biofunctional molecules that possess biomimetic properties and the ability to undergo spontaneous restructuring [117]. The crucial qualities of natural polymers include bioactivity, biocompatibility, 3D geometry, antigenicity, non-toxic byproducts of biodegradation, and inherent structural similarity [100].

Proteins and polysaccharides are, predictably, among the most studied materials as bioengineered scaffolds [100]. Because of their unique features (e.g., antibacterial activity), polysaccharides such as dextran [97], chitosan [102], and hyaluronic acid [103] are widely investigated for cutaneous wound healing (Table 1). Since natural polymers include many different types of active functional groups, they may be easily modified, chemically or biologically, to acquire additional desired functionalities, such as accelerated wound healing.

Generally, natural polymers are involved in biochemical and physiological reactions, rebuilding of the extracellular matrix, cell signaling, and other processes associated with wound healing. Collagen and its subproduct gelatin influence key steps of the wound healing process, such as hemostasis, inflammation, and angiogenesis [53]. At the injury site, collagen induces platelet activation and aggregation and fibrin clot deposition. In the inflammatory stage of wound healing, immune cell activation stimulates the secretion of proinflammatory cytokines and produces the migration of fibroblasts, epithelial, and endothelial cells. The balance between collagen depositions induced by fibroblasts and collagen degradation, and its released fragments that promote fibroblast proliferation and synthesis of growth factors, leads to angiogenesis and re-epithelialization, remodeling of the extracellular matrix, and an increase in the mechanical properties [161]. Keratins are involved in the proliferation of the keratinocytes and the conservation of their integrity in the epithelium [162]. Some keratins support the phosphorylation of STAT3 and its transport to the cell nucleus and increase the translation of D1 cyclin that is necessary for the proper proliferation of keratinocytes [163]. In acute wounds, keratinocytes migrate from the wound margins, proliferating and releasing cytokines to initiate tissue response. However, in diabetic patients, chronic wounds can be produced, such as ulcers, and keratinocytes’ abilities to migrate are restricted, leaving the wound healing process incomplete [164].

Hyaluronic acid has several biological functions, such as skin moisturization and anti-wrinkle effects that support the natural healing process. Additionally, numerous in vitro and in vivo studies revealed the wound healing ability of HA by enhancing mesenchymal and epithelial cell migration and differentiation, as well as improving angiogenesis and collagen deposition [165,166].

Chitosan, a chitin derivative, is an effective hemostatic agent that accelerates wound healing by improving the functions of inflammatory cells, inhibiting the activation of NF-κB (Nuclear factor kappa-B) and lipopolysaccharide (LPS) binding, reducing the production of pro-inflammatory cytokines, and inhibiting tumor necrosis factor-α (TNF-α) [167]. The hemostatic property of chitosan is related to the presence of positive charges in the structure that can interact with the blood’s negatively charged cell membrane, stimulating platelet adhesion, activation, and aggregation at sites of vascular injury [168]. Other polysaccharides, including xanthan gum and gellan gum [156], chemically modified or combined with proteins, have been extensively tested as regulators of hydrophobicity; they are involved in moisturizing environments and regulating cell attachment and proliferation in wound healing [169].

Proteins and polysaccharides have their own variety of strengths and downsides. Some drawbacks associated with collagenic materials include their low stability under high concentrations of proteolytic enzymes and oxidative stress conditions [170]. Chitosan-based scaffolds present challenges regarding acidity exacerbating irradiated tissue, and xanthan gum or gellan gum application is restricted by their very low biodegradability [171,172]. From all studied biopolymers, collagen and chitosan are the most used protein and polysaccharide, respectively, due to their hemostatic and biocompatible properties, while dextran and alginates remain underexplored in clinical scenarios, due to their lower mechanical stability. In order to take advantage of both proteins and polysaccharides’ features, hybrid materials are currently being investigated, leading to their widespread use in the field of cutaneous wound healing [55].

#### 3.2.2. Synthetic Polymers—Partners for Natural Polymers in Wound Healing?

**Figure 5:**
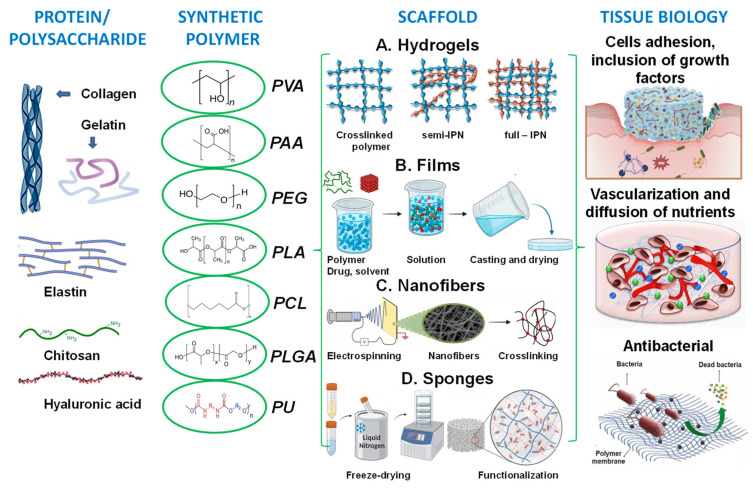
Proteins/polysaccharides and synthetic polymers for wound healing and skin tissue engineering: (**A**) hydrogels with various architectures [176]; (**B**) continuous films [177]; (**C**) nanofibers [178,179]; (**D**) sponges [170,180].

Synthetic polymers, as versatile materials, some of which have favorable biological and physical features, are extensively explored in wound healing applications [107,108]. Compared to natural polymers, synthetic polymers have the benefit of being produced and altered in a controlled manner, have consistent and uniform physico-chemical characteristics, as well as stability and easy maneuverability [173,174]. They lack contaminants, often possess mechanical stability, undergo regulated degradation, and can be processed as architectures that stimulate the cells’ adhesion, proliferation, and differentiation [114]. However, they are physiologically inactive and do not provide a therapeutic benefit as do natural polymers. Synthetic polymers commonly employed in wound healing applications are hydrophobic and hydrophilic and these antagonist characteristics are exploited to regulate the behavior in biological environment (Figure 5); their use in tissue engineering is associated with a targeted tissue, particularly its mechanical properties and rate of biodegradation and gradual replacement of the scaffold by the newly formed tissue [175].

The chemical composition of polymeric scaffolds ultimately governs a variety of characteristics. Table 2 depicts synthetic polymers tested in wound healing applications, their structure and properties, and proteins or polysaccharides as partners in medical applications. Poly (ε-caprolactone) is a biodegradable synthetic polymer and an aliphatic polyester that includes repeated units of hexanoate and is used in the preparation of scaffolds for wound repair and bone tissue regeneration due to its peculiar mechanical properties, biodegradable nature, as well as its miscibility with several polymers [181]. PCL degradation occurs due to the activity of the enzyme lipase, an enzyme released by cells in interstitial fluid that is able to break PCL’s ester bonds; the degradation rate is influenced by the pH of the medium, and 6-hydroxycaproic acid is released as a breakdown product. It was demonstrated that 6-hydroxycaproic acid is taken by cells, undergoes 2-β-oxidation for the formation of 3-acetyl CoA molecules, which are metabolized in the citric acid cycle, and, finally, is eliminated from the body via renal excretion [22,42]. One major drawback associated with PCL is its hydrophobic nature that hampers the wound repair application potential. However, several studies reported prompt wound repair in the presence of composite scaffolds, including PCL. Furthermore, cellular adhesion properties in PCL scaffolds can be enhanced by employing modification techniques such as gamma irradiation or plasma treatment [182,183].

Poly (ethylene succinate) (PES) and poly (butylene succinate) (PBS) are polyesters that are produced via a petrochemical route or biomass-based raw materials. The polymers are biodegradable and biocompatible, and can be easily tailored for different applications, including wound healing. Wound dressings based on PES/PBS are designed with controllable porosity and a high surface area-to-volume ratio that closely resembles the architecture of the natural extracellular matrix (ECM), allowing for moisture retention, hemostasis, removal of exudates, and cell proliferation. Poor mechanical properties and low biodegradation rate due to the polymer’s high ability to crystallize are often modified by co-polymerization with different monomers and have been explored to design cell-friendly architectures [219].

Polyurethanes (PUs) are biocompatible hydrophobic synthetic polymers frequently used for wound dressings because of their barrier properties and oxygen permeability. PUs are also thermoplastic polymers with robust mechanical properties, suitable for various applications, including wound healing and tissue engineering [225]. Dressings based on PUs promote wound healing by reducing leakage via a highly absorbent core that stimulates exudate retention. Such dressing may also prevent infection through a barrier function that blocks liquids and bacteria. The dressings are easy to lift and remove without pain, protecting the skin against trauma and reducing pressure on the wound area. Coatings with collagen or collagen-based peptides on polyurethane meshwork increase the cells’ adhesion and enhance the biocompatibility of the tissue [226].

A few synthetic polymers, like poly (caprolactone) and poly (ethylene succinate) (PES)/poly (buthylene succinate) (PBS), undergo degradation in the presence of bacterial enzymes. This property can be exploited to fabricate smart dressings, wherein the presence of bacterial infection will govern the release of the drug from electrospun nanofibers [227] or bacteria-responsive dressings [231].

Surface features play a significant role since they dictate polymer interactions with cells and proteins; these features include wettability, swelling ability, electrostatic effects, hydrolytic degradation, elasticity, and shape [232]. They have an impact on the neighboring interfacial environment, which facilitates the interactions of proteins/cells on the material’s surface. The interaction between cells and polymers is closely linked to the nature of the functional groups present on the scaffolds’ superficial layers. In most cases, the synthetic polymers having cationic monomers permit electrostatic interactions with negatively charged cell membrane surface; meanwhile, anionic monomers need additional functional materials for their biological activity, like proteins or peptides [233]. Disadvantages in using synthetic polymers are associated with their biodegradation speed and the degradation byproducts in biological medium, including in skin hypoxic wounds, which can negatively influence the wound healing processes by inducing inflammation or immunorejection [234]. Synergistic combinations can be designed with natural and synthetic polymers, and some key performance characteristics related to radiation-induced wound healing are exemplified in Table 3.

### 3.3. Scaffolding Polymers for Wound Healing Applications

The polymers are designed and shaped as microstructures and complex architecture for wound healing, as a supportive substrate facilitating the transport of cells and therapeutic substances [227]. Recent functional wound dressings, such as hydrogels, hydrocolloids, nanofibers, and foam dressings, have demonstrated successful outcomes for the management of radiation-induced injuries [231]. Woven dressings, porous scaffolds, 3D printing materials, nanofibers, hydrogels, and other types of architectures are available at this point to aid wounds in healing [96]. Hydrogels demonstrated significant benefits in the treatment of radiation-induced damages, evaluated in several studies; encouraging results have been obtained due to their superior mechanical and biochemical properties (such as adhesiveness, antibacterial, and antioxidant capacities) [223,232]. Hydrogels, on the other hand, with high water absorbance ability and 3D mesh structure, with exceptional moisture retention and air permeability, offer an extensive adaptability to particular wound healing stages and complications [235].

**Figure 6:**
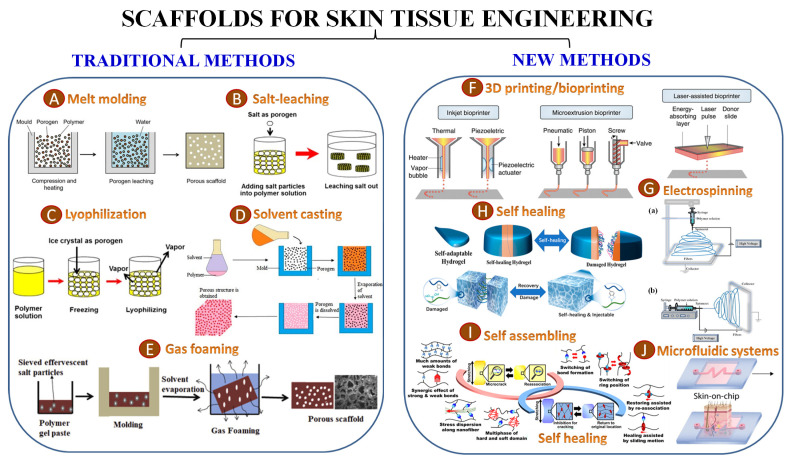
Fabrication of scaffolds for skin tissue engineering: (**A**) melt molding [238]; (**B**) salt leaching [239]; (**C**) lyophilization [240]; (**D**) solvent casting [241]; (**E**) gas forming [242]; (**F**) 3D printing/bioprinting [243]; (**G**) electrospinning ((**a**) vertical; (**b**) horizontal) [244]; (**H**) self-healing [245]; (**I**) self assembling [246]; (**J**) microfluidic systems [247].

The selection of the polymer design and the processing method of the wound healing stimulative architecture are correlated with several requirements of the application: (i) the delivery and retention of cells and biochemical factors; (ii) the facilitation of cell attachment and migration; (iii) the flowing of essential cell nutrients and released products; (iv) the influence on the cell behavior through mechanical and biological stimuli; (v) to recreates an ECM-like microenvironment [236]. Traditional procedures involve the use of several techniques, such as solvent-casting and particulate-leaching, based on polymer solutions mixed with salt particles of certain diameters (Figure 6). After solvent evaporation, the material is submerged in water, and the salt particles dissolve, resulting in a porous structure [237].

Gas foaming is a process that shapes biodegradable polymers at high pressures using gas-foaming agents (or blowing agents), such as CO_2_, nitrogen, water, or fluoroform. The polymers reach a saturation level, and gas bubbles, generally between 100 and 500 μm, are generated and expanded inside the polymeric matrix. Furthermore, phase separation involves a fast reduction in the temperature of a polymer solution and its division into two distinct phases: one phase rich in polymer (undergoes solidification) and another phase with low polymer concentration (eliminated, leading to a porous polymer network with excellent permeability); the solubilized amount of polymer represents an effective tool to tune the foam density and pore morphology [248].

Lyophilization, commonly named freeze drying, provides a technique for manufacturing polymeric micro/macroporous scaffolds. The procedure consists of polymer solution cooling to a precise temperature, resulting in the solidification of all constituents. During the process of freezing, the solvent undergoes a transformation to ice crystals, and polymer molecules connect to each other and gather within the small gaps between the crystals; the solvent is removed by sublimation of the solvent, leaving a desiccated polymer scaffold with a high interconnected microstructure [249]. The porosity of the scaffolds depends on the concentration of the polymer solution, while the freezing temperatures impact the distribution of pore diameters. This approach is not only used to create porous scaffolds, but also to process biological molecule-including samples, in order to protect their bioactivities [250].

Electrospinning technology, 3D bioprinting technology, microfluidic technology, and stem cell technology are new technologies implemented in the production of bioengineered scaffolds [103]. Hence, bioactive materials are a promising therapeutic tool that improve upon the drawbacks of current methods, change wound microenvironments, and trigger various biological, biochemical, and physical factors; they are produced and regulated by endogenous biomediators, exogenous drugs, and external environment, shifting the healing process in the desired direction [251].

Electrospinning employs the electro-hydrodynamic processing of polymeric solutions and produces nanofibers. A basic configuration requires an injection pump, a syringe equipped with a needle, a high voltage power supply, and a collection plate [252]. Generating an electric field between the needle tip and the collecting plate, an electrically charged stream of polymer solution is produced; it flows towards the collector and forms fibers [253]. The approach has been thoroughly investigated in the field of tissue engineering due to the tenability of nanofibers in terms of size and spatial organization, and excellent biological response to cells and tissue formation. Moreover, simultaneous electrospinning of multiple polymeric solutions produces hybrid scaffolds and permits the fine-tuning of the scaffold’s mechanical, chemical, and biological properties [254].

Recently, 3D printing, also known as additive manufacturing, has been extensively tested and applied in the field of TE [255]. This computer-aided design (CAD) technique utilizes a layer-by-layer procedure to produce architectures with intricate structures by adding materials such as ceramics, powders, polymers, metals, or liquids in a systematic manner, following a bottom-up approach [256]. Several 3D printing processes exist, including (i) laser-based 3D printing stereolithography (SLA), selective laser sintering (SLS), and digital laser printing (DLP), (ii) extrusion-based 3D printing includes fused deposition modeling (FDM) [257,258], (iii) ink-based 3D printing specifically involves ink jet printing (IJP), and aerosol jet printing (AJP) [259]. Extrusion-based 3D printing is widely recognized as the most prevalent printing technology and uses a wide range of polymeric ink composites [260].

Three-dimensional printing significantly contrasts with conventional scaffold fabrication techniques by offering precise control of the material deposition and cellular arrangement inside the printed structure, hence enabling the development of complex, customized tissues [261]. The primary benefit of the 3D printing technique lies in its ability to produce 3D architectures that closely replicate the microenvironments of tissues and organs, thereby properly reflecting human physiology [262]. The development of 3D printing has led to significant advances in scaffold design and the ability to accurately mimic the EMC. The method produces tailored complex porous scaffolds with precise manipulation of spatial geometry, microarchitecture, surface-to-volume ratio, and porosity. Three-dimensional printing offers significant advantages in the wound healing process, such as customized dressings that enhance ventilation and moisture regulation, thereby creating an optimal healing environment. It also aids in reducing scarring, which is particularly advantageous for aesthetically sensitive wounds or those exhibiting hypertrophic scarring [263]. Three-dimensionally (bio)printed scaffolds featuring interconnected pores and extensive surface areas facilitate cell attachment, proliferation, intercellular interactions, and the exchange of gases and nutrients, presenting a significant advantage over conventional solvent casting, phase separation, and melt molding methods [264,265]. The application of 3D bioprinting in the field of ADSC-based therapies contributes to the reproduction of the human tissue complexity and represents a significant step toward organ-level functional 3D tissue scaffolds [266,267]. Fu and collab. prepared bioinks based on human adipose tissue decellularized ECM, methacrylated gelatin, and methacrylated hyaluronic acid, loaded with adipose-derived stem cells (ADSCs) and revealed, in a mouse model (full-thickness injury), a complete wound closure, increased deposition of collagen III, and neo-vessel formation after bioprinted architecture application [268]. Roshangar and collab. tested ADSCs seeded into 3D bioprinter-derived gel scaffold based on collagen and alginate, layer-by-layer arranged, in a full-thickness burn rat model and observed a faster epithelization and a multi-layered epidermis with the onset of cornification for a scaffold with an ADSC group, demonstrating the bioengineered scaffold’s potential in skin repair [269].

All these scaffolds need careful sterilization in conditions that do not change the structure and morphology and do not impact the interactions with biological molecules, cells, or tissues. Sterilizing methods include UV and gamma irradiation, heat-based ethylene oxide, immersion in ethanol solution, and argon plasma [270]. The combination of scaffolds with biological molecules (antibacterial, antitumoral) or magnetic moieties is tested in order to stimulate complex mechanisms of action in the human body [271,272].

Despite progress in 3D bioprinting, some limits for the clinical application of biofabricated skin grafts are present. To reach a standardized clinical application, the complexity of human skin tissue needs to be approached, and the challenges to be solved include cell survival during the printing process, the proper cellular nutritional and oxygen supply during and after bioprinting process, a final structural arrangement suitable for proper vessel ingrowth, a lower period for graft vascularization, in vitro and in vivo, etc. Moreover, the reproducibility of the 3D bioprinting is not fully optimized yet, and bioprinted skin grafts are still limited, with safety and legal issues in the clinical use and their standardized application [273].

### 3.4. ADSCs in Skin Tissue Engineering

The advancement in treatment for different kinds of wounds is driven by modern regenerative medicine and tissue engineering, and stem cells are extensively tested [274]. In particular, mesenchymal stem cells (MSCs), which are described as adult native cells, have been considered, based on their capacity to develop in vitro different tissues, including cartilage, bone, and adipose cells, with all exhibiting anti-inflammatory, trophic, paracrine, and immunomodulatory properties [275].

According to studies, the production of adipose-derived stem cells (ADSCs) is 40 times greater than that of bone marrow mesenchymal stem cells (BMSCs), and they can preserve a normal diploid karyotype for 100 generations of culture [276]. Because multipotent ADSCs can differentiate into a variety of tri-germline cells, such as osteocytes, adipocytes, neuronal cells, vascular endothelial cells, cardiomyocytes, pancreatic cells, and hepatocytes, the use of adipose tissue and its cellular products serves as a paradigm for tissue regeneration and cellular restoration [277]. Currently, scientists are concentrating on using such cells to heal persistent wounds [278]. These cells can be added as a cell suspension and injected directly into the wound in extracellular vesicles [279] alongside a compatible carrier or a complex 3D skin scaffold [280,281].

ADSCs are accessible in large quantities, are very simple to harvest from liposuctions carried out under local anesthesia, may maintain their phenotype and plasticity after a prolonged in vitro culture, possess a low immunogenicity, and are demonstrated to be clinically safe, making them an ideal candidate for tissue engineering [282,283]. Moreover, they have received considerable validation of their pluripotency, proliferative performance, and limited donor morbidity [284]. ADSCs are a source of various growth factors, chemokines, cytokines, and paracrine molecules, a complex of biological molecules that promote survival of the cells, modulate the specific inflammatory reaction, and thus enhance the regenerative processes at the injured tissue [285,286]. Chronic radiation skin damage has also benefited from ADSCs. Numerous studies have examined the role of stem cells in wound healing (Table 4), particularly in radiation-induced skin damage [287,288,289,290,291,292,293,294].

The majority of cutaneous radiation damage treatment methods now focus on supportive wound care [295].

### 3.5. ADSCs and Protein/Polysaccharide-Based Scaffolds for Wound Healing

Recently, multiple studies showed that a combination of scaffolds and ADSCs treatment improves wound healing outcomes compared to ADSCs alone [296,297,298,299,300,301].

A biocompatible 3D microenvironment with higher cell-to-cell and cell-to-matrix connections may be produced by the incorporation of ADSCs into scaffolds, simulating an in vivo environment more precisely [302]. ADSCs may be distributed across the whole surface of the wound in a physically solid scaffold, which enhances their impact on the healing process [303].

ADSCs have been studied on wounds paired with a variety of scaffolds, including chitosan [304], fibrin [305], hyaluronic acid [306], collagen sponge [307], collagen peptide scaffolds [308], decellularized tissues [309,310], atelocollagen [311,312], amniotic membranes [313], platelet gels [314], and combinations with different scaffolds [315,316].

Currently, the efficacy of adipose-derived stem cell-based scaffolds in radiation-induced wound healing has not been well defined, and, despite the large number of ADSC–polymer architectures developed, only a limited number of preclinical studies have reported the use of different ADSC therapies combined with bioengineered scaffolds to promote wound healing in post radiotherapy defects. In all these studies, collagen-based scaffolds have been evaluated, and they are summarized in Table 5.

**Figure 7:**
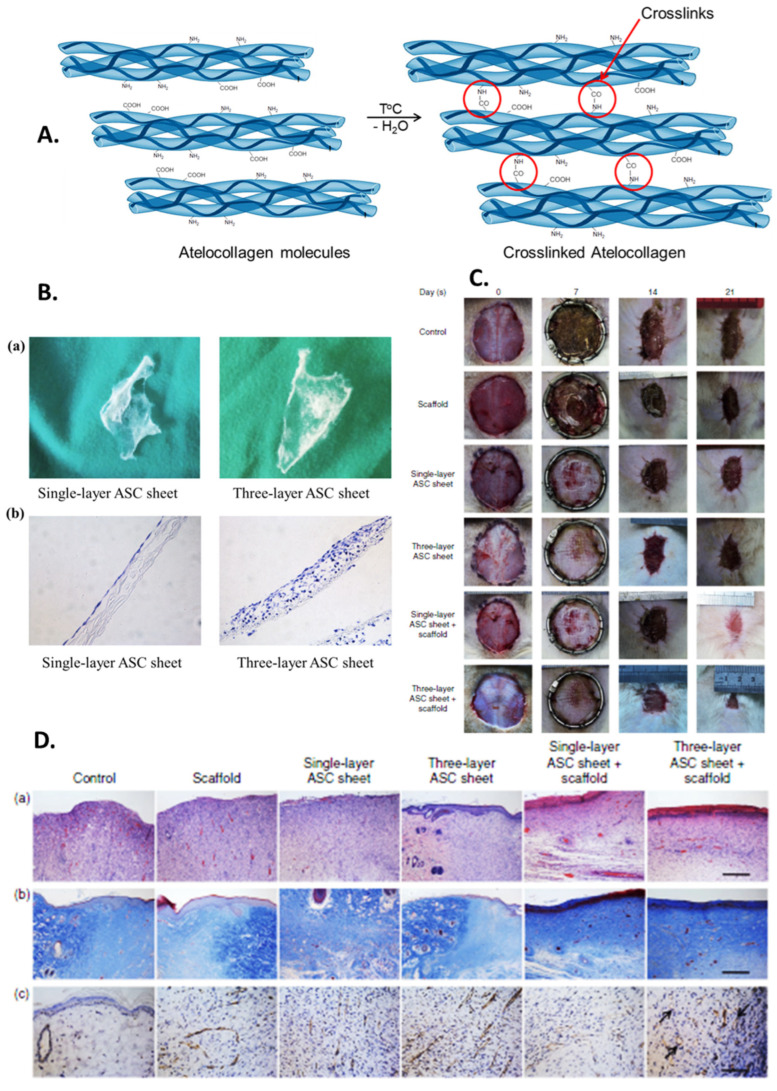
(**A**). Atelocollagen macromolecules crosslinking via EDC method. (**B**) (**a**) Representative macroscopic view of single-layer ADSC sheet (**left**) and three-layer ADSC sheet (**right**) (200×). (**b**) H&E staining of single-layer ADSC sheet (**left**) and three-layer ADSC sheet (**right**) (200). (**C**) Representative macroscopic view of wound repair. (**D**) Histological and immunohistological analysis of wound sections: (**a**) H&E staining of wound sections (100×); (**b**) Masson trichrome staing of wound sections (100×); (**c**) Immunohistochemical staining specific for CD31 (100×). Adapted from [312].

The study developed by Zhang et al. (Figure 7) was focused on the application of three-layer ADSC sheets combined with atelocollagen scaffold to improve wound healing in mice.

An atelocollagen-based scaffold was obtained by crosslinking via the EDC method (Figure 7A), seeded with ADSCs, and tested as a single-layer ADSC-seeded sheet or a three-layer ADSC-seeded sheet (Figure 7B) on radiation-induced burns on dorsal skin. The authors measured wound sizes (Figure 7C), observed cell proliferation, and explored the mechanisms behind the improved wound healing. They assessed wound size reduction, cell proliferation, inflammation, angiogenesis, and collagen formation. In terms of investigated mechanisms and clinical implications, the authors evaluated the ADSCs’ influence on wound healing through cell proliferation, reduction of inflammation (e.g., IL-1b expression), promotion of angiogenesis (e.g., VEGF expression), and collagen formation. The histological data and immunohistochemical assays (Figure 7D) suggest that ADSCs and atelocollagen scaffolds can work synergistically for skin wound regeneration.

**Figure 8:**
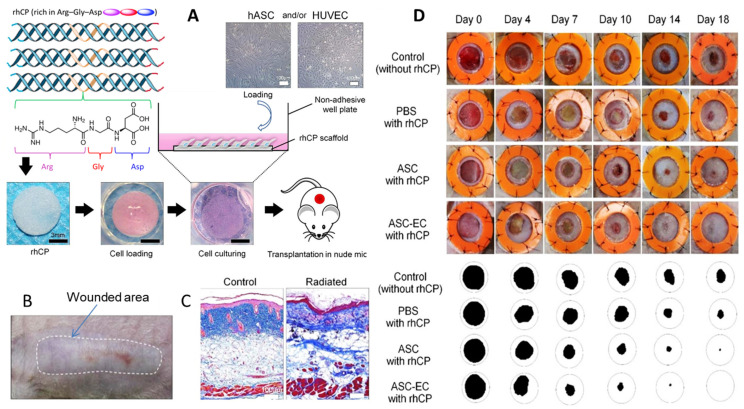
(**A**) Recombinant collagen type-I peptide macromolecular structure, scaffold preparation, and transplantation methods; (**B**) appearance before wound creation. This model underwent 20 Gy of radiation on the back 4 weeks ago and resulted in macroscopic tissue atrophy and fibrosis. (**C**) Histological analysis revealed fibrosis and the disappearance of cutaneous appendages. (**D**) Representative photos of cutaneous wounds (6 mm) created on the irradiated dorsal areas of mice (*n* = 3 in each group), with 18-day follow-up. Ulcerated surface areas were determined via digital software (Photoshop CS6; Adobe Systems, San Jose, CA, USA) (**B**–**D**). Adapted from [308].

Maskito T. et al. [308] discuss wound healing in irradiated tissue models in nude mice. They used rhCP (recombinant human collagen type I) scaffolds, prepared as a 3D sponge, seeded with human adipose-derived stem cells (hADSCs) and human umbilical vein endothelial cells (HUVECs) (Figure 8A), and explored how the rhCP scaffold, hADSCs, and HUVECs collectively contributed to wound healing. They hypothesized a mechanism through integrin binding and growth factor signaling pathways and observed wound closure and tissue regeneration (Figure 8B–D), suggesting the potential for clinical use. Using microarray assays, the authors evaluated the gene expression up-regulation related to cell cycle and wound healing (especially extracellular matrix), and concluded that rhCP induces stem cell activation-like responses such as proliferation, migration, and ECM production by hADSCs.

Pathway analyses revealed gene expression patterns for inflammatory suppression, cell growth promotion, and increased signals by growth factors/cytokines, including VEGF, HGF, and TGF-β1. The authors suggested that the signaling pathways of the VEGF receptor were activated by the rhCP containing multiple RGD motifs, and rhCP worked as a functional cell scaffold, which enhances hADSC wound healing potential, stimulating the hADSCs’ activity and their biological activity in wounds. Some limitations of their studies include the detailed mechanism of rhCP stimulation on cell function and the relation with cell surface receptors and signaling pathways activated, based on the small number of arrayed samples.

**Figure 9:**
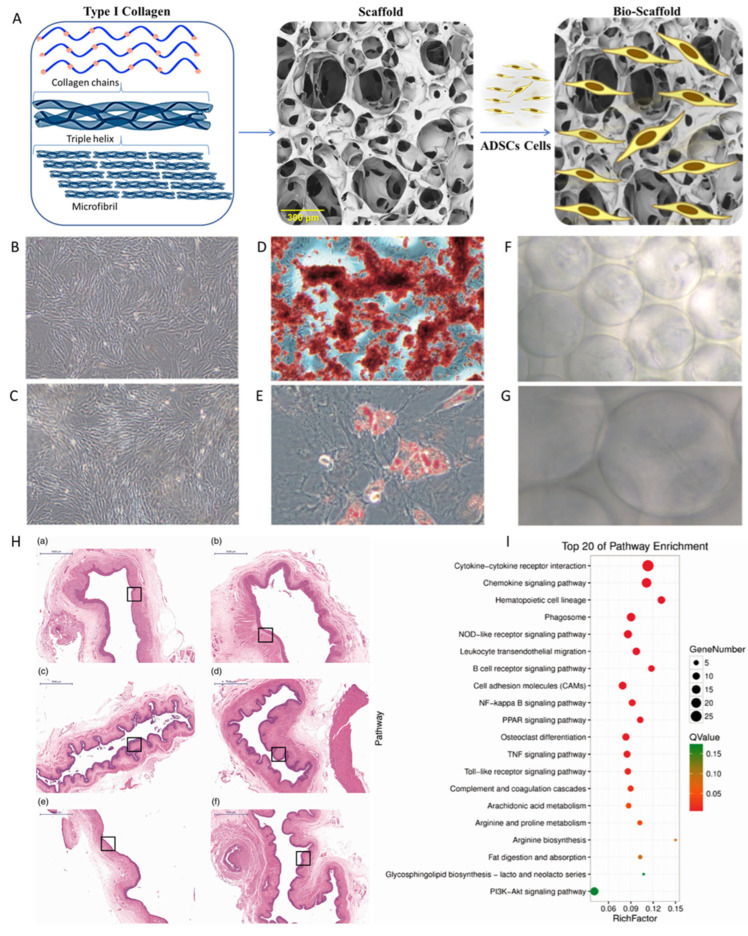
(**A**). Three-dimensional network structure with micron-sized holes made of type I collagen. (**B**,**C**) Isolation and identification of ADSCs (200×). The ADSCs exhibited typical fibroblastic morphology. (**D**,**E**) ADSCs differentiate into osteoblasts and adipocytes (×400). (**F**,**G**) Spindle cells within the scaffold were faintly visible, and the growth state was good and stable. ((**F**) ×400). The collagen structure is visible. ((**G**), ×1000). Spindle cells within the collagen scaffold. (**H**) Hematoxylin and eosin staining data: (**a**) Injection of PBS into irradiated site; (**b**) injection of protein scaffold into irradiated site; (**c**) injection of ADSCs into irradiated site; (**d**) injection of complex into irradiated site; (**e**) untreated irradiated rats; (**f**) blank control group. *n* = 5 rats per group. Scale bar  =  1000 μm. Squares indicate the studied areas. (**I**) The NF-κB pathway was enriched for some up-regulated genes. The diameter represents the number of differentially expressed genes; the wider the diameter, the greater the number. Red represents statistical significance (adapted from reference [316]).

Ye and collab [316] focused on the healing of radiation-induced vaginal injuries in rats using ADSCs-protein scaffold complexes. They cultured adipose-derived stem cells within a 3D protein scaffold made of type I collagen (Figure 9A–G) and transplanted them into rats with vaginal injuries. Histological analyses were used to assess regeneration of the vaginal epithelium, and the examination for RNA completed the study in order to explain the therapeutic mechanism of the cell–collagen constructs (Figure 9H,I).

The authors concluded that ADSCs may mediate repair through the activation of the NF-kB signaling pathway, which plays a major role in controlling inflammation. It was demonstrated that NF-jB activation is involved in the transcriptional control of acute and chronic inflammation, and the cell-scaffold system induces anti-inflammatory effects through this pathway. Investigation of the healing mechanism of ADSCs using RNA sequencing confirmed that ADSC-based bioscaffold contributes to the repair process by activating the expression of the NF-jB signaling pathway.

Additionally, the study examined the expression of mucin MUC1 as a marker of epithelial cell function, and delays in injury healing for untreated animals were observed, with some not healing at all, as well as prolonged *PCNA* (proliferating cell nuclear antigen) and MUC1. The study did not find a significant difference between the ADSC–collagen-treated group and the negative control group in terms of *PCNA* expression. The study suggests that ADSCs-protein scaffold complex could be a useful therapeutic approach for patients suffering from vaginal injuries following radiotherapy, addressing a specific clinical need.

**Figure 10:**
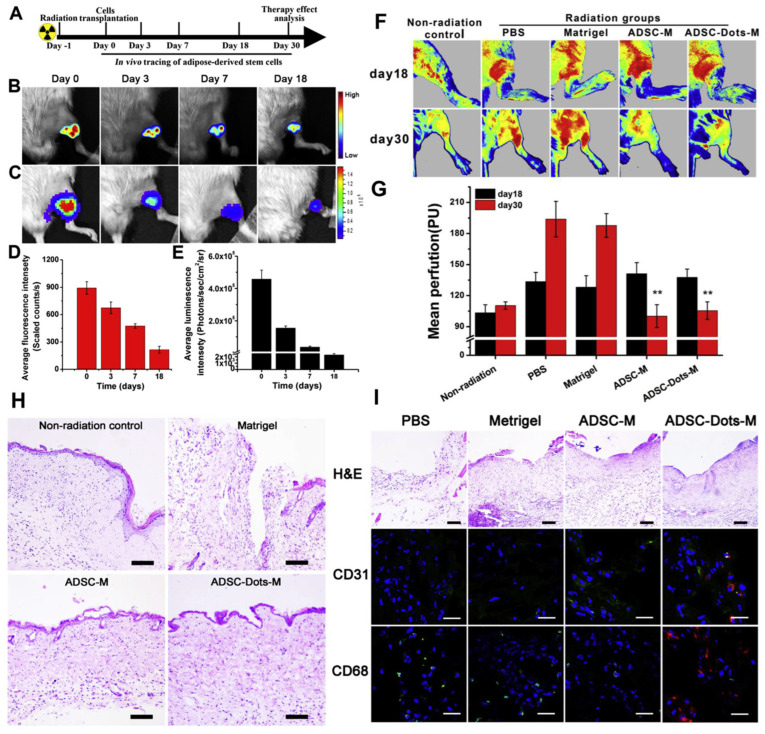
In vivo experiments on adipose-derived stem cells (ADSCs) included in collagen matrix, in a mouse model with radiation-induced skin injury. (**A**) Time schedule of in vivo ADSCs tracking experiments. (**B**) Representative time-dependent fluorescence images of radiation injury-bearing mice via AIE dots. (**C**) Time-dependent in vivo bioluminescence images of the same mouse in (**B**). Quantitative analysis of fluorescence intensity (**D**) and luminescence intensity (**E**) in the injury region shown in (**B**,**C**), respectively. (**F**) Laser Doppler perfusion images of limb lesions of radiation injury-bearing mice at 18 days and 30 days post-transplantation, and (**G**) quantitative analysis of perfusion at 18 days and 30 days after ADSCs transplantation; ** *p* < 0.01. (**H**) H&E staining images of limb lesions of radiation injury-bearing mice. Scale bars, 100 μm. (**I**) H&E staining images and immunostaining images against CD31 and CD68 of skin lesions of radiation injury-bearing mice at 30 days post-transplantation. Scale bars: 100 μm for H&E images; 25 μm for immunostaining images. (Adapted from [317]).

Yang and collab [317] reported, in their study, seeing the fate and regenerative mechanism of adipose-derived stem cells (ADSCs) included in a collagen matrix in a mouse model with radiation-induced skin injury (Figure 10A—the study design). Fluorescent nanoparticles were prepared and internalized by ADSCs as an effective fluorescent cell tracker to precisely follow the cells and their behavior in an animal model (Figure 10B–G). Labeled ADSCs were encapsulated into Matrigel and then injected into the injured sites (obtained on the left hind limb skin by exposure at 40 Gy of X-ray, using a dose rate of 2 Gy/min for 20 min). Histology and immunohistochemistry assays (Figure 10H,I), as well as cell tracking tests, were performed. Under tested conditions, in all radiation-induced lesions, a more inflammatory reaction than the non-radiated group was observed 18 days after ADSCs transplantation. ADSC transplantation in the presence of Matrigel demonstrated a significant decrease in inflammation (about 62% of PBS and Matrigel groups). Histology and immunohistochemistry data indicated that cell presence boosts the skin healing of radiation lesions, and more blood vessels and fewer macrophages were found in the wounded area.

The authors concluded that transplanted ADSCs in radiation-induced skin injury can be noninvasively and precisely analyzed by tracing with fluorescent nanoparticles, without an unfavorable impact on the regeneration effect of labeled adipose-derived stem cells. Meanwhile, this study revealed the great benefits of using complex cells in the repair of radiation-induced skin injury.

These articles collectively contribute to the field of radiation-induced wound healing, supporting the positive benefits from a combined ADSC and polymeric scaffold therapy by exploring different cell types, scaffold materials, and experimental models to address various aspects of tissue healing and regeneration. While preclinical studies share the common goal of promoting tissue repair, they approach the topic from different perspectives and have distinct clinical applications and limitations.

## 4. Clinical Translation of ADCS-Based Bioengineered Scaffolds

The FDA has approved multiple skin substitutes for particular applications, including the treatment of burns, diabetic foot ulcers, and chronic wounds, such as Apligraf, Dermagraft, Integra, StrataGraft, and Acellular Dermal Matrices [318]. Apligraf is a composite skin substitute based on human epidermal and dermal cells on a collagen scaffold, approved for diabetic foot ulcers [319]. Dermagraft is another composite skin substitute, using cryopreserved human fibroblasts on a collagen scaffold, also approved for diabetic foot ulcers [320]. StrataGraft is an FDA-approved skin tissue substitute made from cultured keratinocytes and dermal fibroblasts, used to promote wound healing [321]. Another approved system, Epicel, is a cultured epidermal autograft used for treating burns, especially those covering more than 75% of the total body surface area. Integra is a skin substitute used for burns, approved through the PMA process [322]. The FDA currently has limited approval of stem cell-derived products for cosmetic medicine and surgery due to insufficient data from both animal and clinical research. Moreover, the clinical trials based on stem cells remain limited due to the complexity of studying the risks to patients, including local reactions at the administration site, rejection responses, migration to other locations, differentiation into unsuitable cell types, inefficacy, and the chance of cancer development [323]. Additionally, due to their intricate culture procedures and low transformation efficiency, the application of induced pluripotent stem cells is still under evaluation. Comprehensive preclinical investigations employing lineage-tracing models and tumor-susceptible animal populations, in conjunction with real-time molecular surveillance in clinical trials, are important in clarifying these risks. In addition to oncogenic risk, donor variability and ethical concerns are very important [324]. From this point of view, for ADSCs derived from a donor, ethical considerations around informed consent and the potential for exploitation must be addressed before the use of extracted cells.

The developed scaffolds for radiotherapy-induced wounds must be engineered to facilitate healing without the interference of radiation therapy and to prevent the introduction of new irritants or obstructing the healing process. Radiotherapy-induced wounds involve a wet healing environment that facilitates cell migration, proliferation, and tissue regeneration. Scaffolds must be engineered to retain moisture while facilitating enough gas exchange to not induce allergic responses, tissue injury, or inflammation [233], and few commercial scaffolds have been particularly developed for the regeneration of radiotherapy-produced wounds. Flaminal^®^ and Flaminal^®^ RT are sophisticated wound care dressings based on hydrogels, formulated to proficiently address the intricate skin responses induced by radiotherapy (radiodermatitis) [325]. Polymem^®^ is a dressing for the regeneration of inflamed, peeling, and moist skin surfaces injured by radiotherapy exposure or proton beam therapy treatment [326]. Based on the hydrogel’s ability to carry the ADSCs and to be processed as 3D architectures, preclinical and clinical tests have to demonstrate the potential of applying some products with ADSCs safely and efficiently in radiation-induced skin wounds.

## 5. Challenges and Future Perspectives

Advances in wound healing treatments are testing the boundaries of medical research. Novel techniques, which range from cutting-edge dressings to stem cell treatments and bioengineered skin replacements, are not only changing chronic wound treatment but also raising the expectations for faster and more efficient tissue repair. As research in this field continues to advance and to evaluate new combinations of materials, cells, and biomolecules, it can be anticipated that radiation-induced wounds that formerly caused considerable obstacles will be more easily managed and healed in the near future.

**Figure 11:**
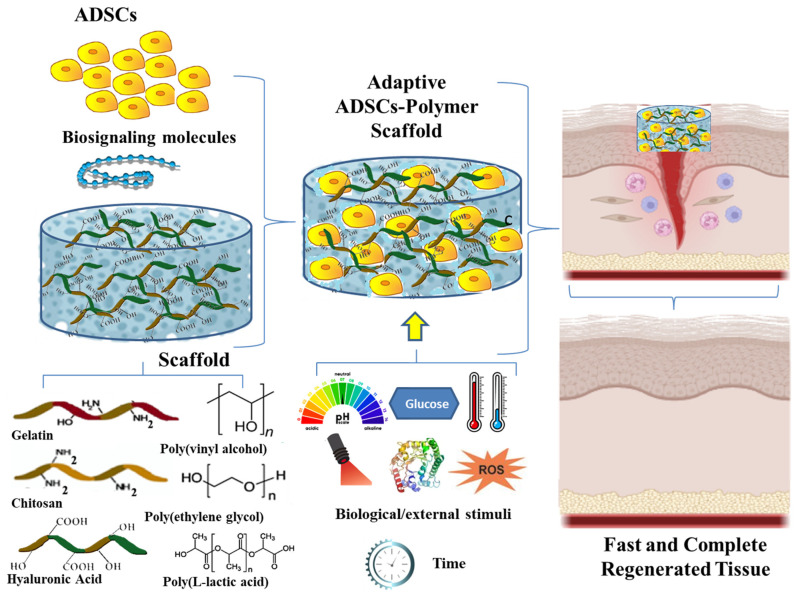
Biological and time-responsive ADSC–proteins/polysaccharides in scaffolds as adaptive architectures for radiation-induced wound healing.

Protein/polysaccharide-based scaffolds and combinations with synthetic molecules, such as hydrogels and nanofibers, micro- and nanoparticles, have displayed impressive outcomes in managing skin injuries, owing to their mechanical and biochemical properties. New time-responsive and biological-responsive scaffolds capitalize on all insights in material engineering; understanding stem cells’ biology and their involvement in wound healing processes are important steps in creating effective bioengineered treatments. The synergy between ADSCs and these scaffolds fosters a microenvironment conducive to tissue repair, allowing for improved cell-to-cell, cell-to-matrix interactions and bioscaffold interaction with healing tissue (Figure 11).

In the quest for more effective wound healing, advancements continue to refine treatments, and advanced bioengineering technologies include hierarchically distributed 3D bioprinted architectures, 4D and 5D bioscaffolds, immuno-engineered pro-regenerative scaffolds, or multi-cell type cultured constructs with predictable bioevolution and interactions.

The diversity of the bioengineered ADSCs-based products should not represent a hindrance to development in this field; on the contrary, there is great potential for future versatile therapies. However, further studies are necessary to standardize the radiation-induced skin injury experimental model, to optimize the method of ADSC extraction and select the scaffold composition and architecture, and to extend the evaluation time in order to obtain accurate results.

Additional clinical studies will undertake balanced risk–benefit considerations with respect to ADSCs’ potential in radiation-induced wound healing treatments. As research in this domain progresses, the possibility of treating difficult wounds through a combination of bioengineered scaffolds and cell-based therapies becomes closer to clinical practice.

## Figures and Tables

**Table 1:** Proteins/polysaccharides and their properties for biomedical applications.

Polymer Chemical Structure	Structural Details	Properties	Ref.
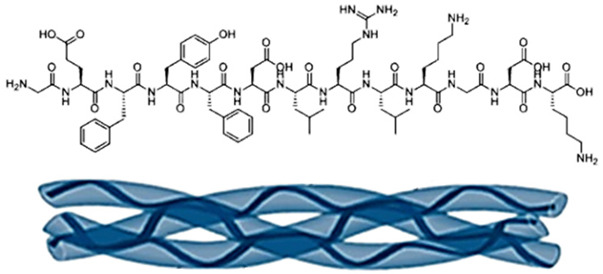 Collagen	Fibrous protein, Contains amino acids, forming a triple helix of elongated fibril.	Low immunogenicity, Excellent biocompatibility, Promote cellular adhesion, migration, and growth.	[118,119,120]
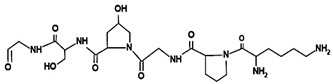 Gelatin	Product of partial hydrolysis of collagen isolated from animal skin, Translucent, colorless, flavorless.	Arginine-Glycine-Aspartic acid (RGD) sequences present in collagen, Promote cellular proliferation, adhesion, and differentiation, Accelerated wound healing mechanism, Gel-forming property.	[121,122,123,124]
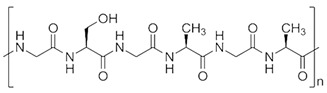 Silk fibroin	Protein, Contains alternate repeating units of hydrophobic and hydrophilic heavy and light chains.	Good biocompatibility with various cell types by promoting their adhesion, proliferation, growth and functionality Gas selective permeability, Antibacterial, UV-protection.	[125,126,127,128]
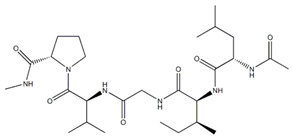 Elastin	Structural protein, A component in the ECMs of connective tissues.	Biocompatible, Biodegradable, Elasticity, Self-assembly, Long-term stability.	[94,129]
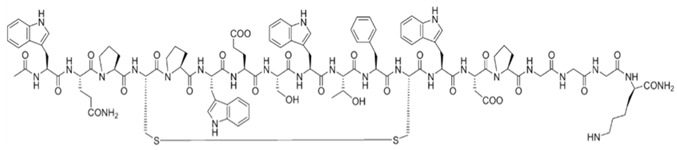 Fibrin	Fibrous, non-globular protein	It has both viscous and elastic characteristics, Mechanically deform-resistant, Biocompatible and biodegradable, Gell-forming ability, Promotes blood clotting and wound healing	[130,131,132]
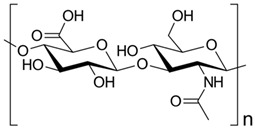 Hyaluronic Acid	Linear polysaccharide, Non-protein glycosaminoglycan with specific physiochemical characteristics.	Non-immunogenic Excellent biocompatibility and hydrophilicity, Regulate cell behaviors and tissue functions, Key macromolecular component of the ECM in most connective tissues.	[133,134,135,136,137]
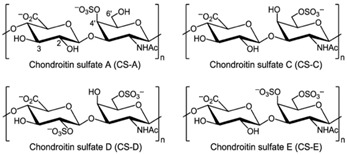 Chondroitin sulphates	Unbranched polysaccharide, Component of ECM.	Antitumoral, Antiviral, Immunomodulatory, Anticoagulant, Antithrombotic properties.	[138,139,140]
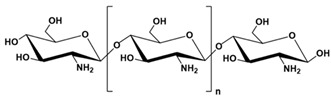 Chitosan	Randomly ordered linear polysaccharide structure with β-(1 → 4)-link and deacetylated D-glucosamine and N-acetyl-D-glucosamine.	Mucoadhesive, Anti-inflammatory, Antioxidant, Antifungal, Antimicrobial, Antihyperglycemic Antitumoral, Wound healing.	[141,142,143]
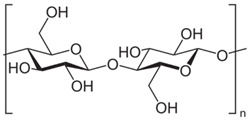 Cellulose	Linear polysaccharide	High purity, Nanofibrillar structure, Biocompatibility, Cells fixation, High water retention and gas exchange, Outstanding mechanical qualities.	[144,145,146]
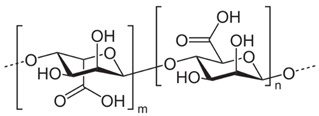 Alginate	A linearly organized anionic biopolymer found in brown algae and bacteria. Contains α-l-guluronic acid (G) and β-d-mannuronic acid (M) residues in linear 1.4-glycosidic linkages.	Reaction with metal ions, Viscos and gel-forming, Dynamical viscoelasticity, Ability to retain water, Ability to enhance monocytes to produce high levels of cytokines (Interleukin-6).	[147,148,149,150]
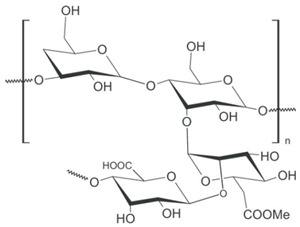 Xanthan Gum	Branched polysaccharide.	Biodegradable, Biocompatible, Superior rheological properties, Mechanical stability, Non-toxic, Thermally stable, Immunological properties, Ability to form stable, transparent, rigid gels at low pH	[151,152,153]
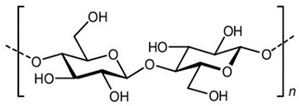 Gellan Gum	Linear, negatively charged polysaccharide.	Good antibacterial activity, Excellent biocompatibility, Biodegradability, Good bioadhesive properties, Non-toxicity, Promote cell migration and attachment, Provide gaseous permeation.	[154,155,156,157]
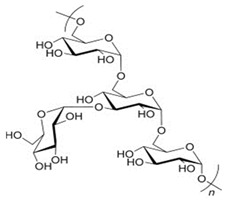 Dextran	Branched polysaccharide.	Biocompatibility, Non-antigenic, Non-immunogenic, Feasible for easy modification with photochemically or thermally crosslinkable groups, Stable under mild acidic/basic conditions.	[158,159,160]

**Table 2:** Synthetic polymers, their properties, and protein/polysaccharide partners in biomedical applications.

Polymer Chemical Structure	Structural Details	Properties	Natural Polymer Used as Partner
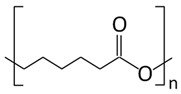 Poly(ε-caprolactone) [184,185,186]	Semicrystalline linear synthesized using ring-opening polymerization of ε-caprolactone.	Can be easily modified, Biocompatible, Hydrophobic, Slowly degraded in vitro in the absence of enzymes.	Collagen [187] Sodium alginate [188] Polysaccharides/ proteins [189]
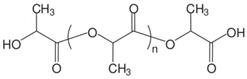 Poly(L-lactic acid) [190,191,192]	Ester-bonded monomer, Assembly of complex structures (branched, star-shaped, grafted).	Thermoplastic aliphatic polyester, Adequate mechanical properties, Tailorable biodegradability, Biomedical applications.	Silk fibroin [193] Hyaluronic acid [194] Cellulose, chitosan [195]
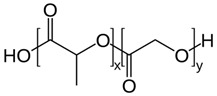 Poly(lactic-co-glycolic acid) [196,197,198,199]	Co-polymers consist of cyclic dimers of glycolic acid and lactic acid.	Good mechanical properties, Biocompatibility, Crystallinity impacts its mechanical strength, swelling, and biodegradation rate.	Chitosan [200] Collagen [201] Cellulose [197] Heparin [202]
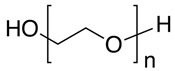 Poly(ethylene glycol) [203,204,205,206]	Semi-crystalline synthesized using low molecular weight polyether monomers generated from ethylene oxide.	Promote collagen deposition, Biocompatibility, Non-immunogenic, Protein resistance ability, Reduce inflammation, Accelerate vascularization.	Alginate [207] Cellulose [208] Hyaluronic acid [209] Collagen [210]
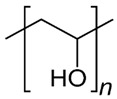 Poly(vinyl alcohol) [211,212,213]	Highly crystalline, synthesized using free-radical polymerization of vinyl acetate.	Non-toxic, Biocompatible, Biodegradable, Mechanical strength, Good hydrophilicity, Thermal stability, Film-forming.	Alginate [214] Chitosan [211,212] Cellulose [213]
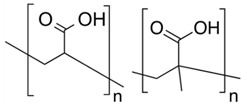 Poly (acrylic acid)/ Poly (methacrylic acid) [215,216]	Synthesized using free radical polymerization method.	Biocompatible, Non-toxic, High swelling capacity.	Chitosan [217] Dextran [218]
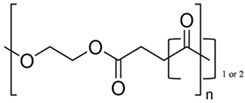 Poly(ethylene succinate) (PES)/Poly(butylene succinate) (PBS) [219,220,221,222]	Dicarboxylic acids, ring-opening polymerization of succinic anhydride with ethylene oxide (PES)/direct esterification of succinic acid with 1.4-butanediol(PBS).	Biocompatible, Biodegradable, Non-toxic products, Mechanical strength, Extrudable and processable as nanofibers.	Proteins/ Polysaccharides [223] Starch [224]
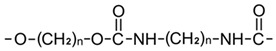 Poly(urethane) (PU) [225,226,227]	Anorganic homopolymer macromolecule composed of carbamate (-O-CO-NH-) linkages.	Biocompatible, Biodegradable, Reduce leakage Blocks liquids and bacteria, Easy to lift and remove without pain.	Collagen [228,229,230]

**Table 3:** Polymers’ properties in radiation-induced wound healing.

Key Performance Features	Natural Polymer	Synthetic Polymer
Hemostasis	Collagen [118], Gelatin [121], Fibrin [131], Chitosan [142], Cellulose [145]	
Anti-inflammatory response	Collagen [118], Alginate [148]	Poly(ethylene glycol) [205]
Immunomodulation	Dextran [159], Xanthan Gum [152]	
Angiogenesis	Collagen [118], Gelatin [121], Hyaluronic acid [134,135]	Poly(ethylene glycol) [205]
Collagen remodeling	Collagen [118], Gelatin [121], Hyaluronic acid [134,135]	
Degradation rate	Chitosan [142], Elastin [129], Hyaluronic acid [135], Xanthan Gum [152], Silk fibroin [126]	Poly(ε-caprolactone) [126] Poly(lactic-co-glycolic acid) [197], Polyurethanes [226]
Mechanical resilience	Hyaluronic acid [135] Xanthan Gum [152], Cellulose [145], Elastin [129],	Poly(vinyl alcohol) [212], Poly(ethylene succinate)/ Poly(butylene succinate) [220], Polyurethanes [226]
Printability	Gelatin [121], Hyaluronic acid [135], Chitosan [142]	Poly(ε-caprolactone) [126], Poly(lactic-co-glycolic acid) [197], Poly(ethylene glycol) [205]

**Table 4:** ADSCs tested on radiation-induced injuries.

ADSCs Origin	Wound Model/ Lesion	Application Conditions	Outcomes	Ref.
Human	In vitro cell culture	Total dose: 20 Gy (single dose of 1 Gy/min, over 20 min)	Reduced inflammation Reduce the HDF apoptosis Increased deposition of ECM	[3]
Inguinal fat pads Sprague–Dawley rats	Sprague–Dawley male rats	A single dose of 50 Gy, 900 cGy/min; Injected 10^6^ ADSCs/0.8 mL of PBS	Growth of the epithelium and muscle Increased blood vessel density Increased dermal thickness of the healed skin	[278]
Peri uterine fat tissue of the rats	rats	Total dose: 30 Gy, single dose of 0.28 Gy/min; 1 × 10^6^ ASCs/300 µL PBS, transplanted locally	Decreasing the wound size Better effect in combination with growth factors	[280]
Groin region of Sprague-Dawley rats	rats	Total dose: 20 Gy, single dose; 1 mL suspension with 3 × 10^6^ ADSCs	Increased flap viability Enhanced blood supply	[292]
Human and mice	male mice	Total dose: 20 Gy (1.51 Gy/min; 1 × 10^6^ ADSCs (2 injections of 50 μL)	Promote reepithelialization and angiogenesis Improved skin blood perfusion and capillary density	[10]
Autologous and allogeneic	mini-pigs	Total dose: 50 Gy (^60^Co gamma source); Injection (5 times)	Reduced local inflammatory injuries Skin healing without necrosis or uncontrollable pain	[9]
Male Sprague-Dawley (SD) rats	rats	Total dose: 90 Gy; 1 × 10^7^ ADSCs (injection within 24 h)	Reduced inflammation, fibrosis, and apoptosis Enhanced sebaceous gland regeneration Downregulated Cathepsin F and downstream pro-apoptotic proteins Upregulated anti-apoptotic proteins	[4]
Guinea pigs	Mature female guinea pigs	Radiation dose: 60 Gy, dose rates: 2 Gy/min; 2 × 10^6^ ADSCs, injection + ultrasound radiation	Wound healed faster Increasing the epithelialization and formation of collagen	[6]
Human, abdominal liposuction	Human female	Therapeutic radiation (75.0 + 35.4 Gy); ~0.15 × 10^7^ ADSCs, injection	Soft texture Increased local blood circulation	[287]

**Table 5:** Combinations of ADSCs and collagen-based scaffolds for radiation-induced wounds, preclinical studies.

Scaffold/Substituents	Animal Model	Wound Model	Radiation Dose	ADSCs Origin/Cell Types	Duration of Evaluation	Outcomes	Ref.
Atelocollagen matrix	mouse	Radiation burn (immediate)—dorsal skin	40 Gy dose at a 25 cm source	Mouse adiposederived MSCs from bilateral groin tissue	21 days	1. Smaller wound sizes 2. Accelerated wound healing 3. Accelerated angiogenesis 4. Increased collagen production 5. Decreased infammation 6. Increased cell profliferation 7. ADSCs upregulate VEGF 8. ADSCs downregulate IL-1β	[312]
Human collagen peptide (rhCP) scaffold	mouse	Radiation burn (2 days)—dorsal skin	20 Gy: a dose of 10 Gy applied twice over successive days	Human adipose-derived stem cells (hADSCs) from liposuction aspirates	4 weeks after irradiation + 18 days post transplantation	1. stem cell activation-like responses such as proliferation, migration and ECM production by hADSCs 2. enhanced cell proliferation 3. wound healing properties 4. accelerating repair of irradiated wounds 5. secretion of trophic factors and immune-modulating capacity	[308]
3D protein scaffold with micron-sized holes made of type I collagen.	rat	Radiation burn on pelvic area—vaginal injury	30 Gy, 25 Gy, and 20 Gy radiation doses	Rats ADSCs from inguinal pads	2 weeks	1. thicker vaginal epithelia, substantial repair 2. NF-jB pathway as a mechanism of tissue repair 3. promoted healing 4. inflammation control 5. potential for clinical use in genitourinary tract reconstruction 6. expression of mucin MUC1 as an epithelial cell function marker	[314]
Matrigel—collagen derived matrix	mouse	Radiation burn on left hind limb skin	40 Gy at a dose rate of 2 Gy/min for 20 min	Mouse adipose-derived stem cells from abdominal and inguinal adipose tissue	30 days	1. decrease in inflammation 2. more CD31-positive blood vessels 3. less CD68-positive macrophages 4. posibility for traking with cuantum dots 5.angiogenetsis effect	[315]

## Abbreviations

The following abbreviations are used in this manuscript:

ADSCs	Adipose-derived stem cells
BMSCs	Bone marrow-derived stem cells
CAD	Computer-aided design
CTGF	Connective tissue growth factor
CRT	Conventional Radiotherapy
DNA	Deoxyribonucleic acid
ECM	Extracellular matrix
HIF1A	Hypoxia inducible factor 1
HSCs	Hematopoietic stem cells
IGRT	Image-Guided Radiation Therapy
IL	Interleukins
IMRT	Intensity-Modulated Radiation Therapy
LPS	Lipopolysaccharide
MMPs	Matrix metalloproteinases
NF-κB	Nuclear factor kappa-B)
PBS	Poly(butylene succinate)
PCL	Poly (ε-caprolactone)
PES	Poly(ethylene succinate)
PUs	Polyurethanes
RTX	Radiotherapy
TGF	Transforming growth factor
TE	Tissue engineering
TIMPs	Tissue inhibitor of metalloproteinases
VEGF	Vascular endothelial growth factor
VCAM	Vascular cell adhesion molecule
